# Ready for 80 more years

**DOI:** 10.5935/1808-8694.20130048

**Published:** 2015-10-04

**Authors:** Henrique Olavo de Olival Costa

*“Age is an issue of mind over matter. If you don't mind, it doesn't matter.” - Mark Twain*

When I think about what the 80 years of the now Brazilian Journal of Otorhinolaryngology, I can't help but think about what Francis Bacon said about aging: “Age appears to be best in four things; old wood best to burn, old wine to drink, old friends to trust, and old authors to read”. We could add that traditional publications are better to cite.

This is so because a journal which managed to keep alive for 80 years not only represents the present moment, but it carries all the intellectual effort of the community that propagated its scientific ideas for this entire period.

We can feel this when we turn our attention towards some of the best known journals in the world: Otolaryngology Head and Neck Surgery, 148 years; Archives of Otolaryngology Head and Neck Surgery, 139 years; Journal of Otology Laryngology, 127 years; Laryngoscope, 123 years and Annals of Otolaryngology Head and Neck Surgery, 121 years.

After all, which should be the role played by a scientific journal? Our journal started as a repository of academic discussions that occurred in the São Paulo Medical Association, in the years 30 of the last century, and it quickly expanded to be the promoter of papers presented by our colleagues in the first Brazilian Otorhinolaryngology Meetings. Later, the so-called “loose” papers began to emerge in its pages and increasingly the papers represented the national ENT community.

However, this role started to be challenged with the advent of institutional graduate programs. Among other requirements, students and professors had to have their scientific studies published in journals of international visibility, so they could achieve important points in the score assigned by CAPES - the institution managing graduate programs in Brazil. Such requirement could be considered a fundamental shift in our journal's trajectory.

Before, more concerned in providing space to Brazilian specialists, the journal felt motivated to be read and to have papers cited by foreign journal authors, gain international status and be inserted in indexation institutions considered by CAPES (i.e, JCR-ISI).

This concern lingered in all national research sectors, and as an initiative of the FAPESP - Research Support Foundation of the State of São Paulo; the Regional Library of Medicine (BIREME) initiated a project aiming at indexing and providing visibility to the main scientific journals of Latin America. The indexation platform was called SciELO (Scientific Index Electronic Library-online) and established similar or superior publishing requirements to the ones of the main indexing agencies of the world. It intended to set a high-enough standard, so that the chosen journals could be competitive vis-à-vis foreign journals.

So much so that, the then Brazilian Society of Otorhinolaryngology, considered this the ideal opportunity for the Brazilian Journal of Otorhinolaryngology (RBORL) to climb some steps in its publishing status.

In order to be accepted in the SciELO, we needed regular bimonthly publications, at least 30 papers published per year and 75% of them had to be original studies. To meet these requirements it was necessary that the entire community, headed by the Brazilian Society of Otorhinolaryngology, would embark in an improvement process.

At that time, there was a strong demand from the Academies of subspecialties to unify individual meetings. We then started the Triological Society Meetings, which alternated with the Brazilian Meeting, and went on to play a relevant scientific role. This was the cue for a stronger stimulation to scientific production. We then started awarding the best papers, and team of revisers started to be part of the Scientific Evaluation Committee in Meetings. In order to have the assessment, the competing papers should be referred to the meeting's Organizing Committee in full and complying with the standards required by the BJORL.

At the time, we had no idea of which would be the impact of these measures. For our satisfaction, the number of papers sent to our journal increased substantially in the subsequent years - since most of the papers sent to the Meeting's contest were ready for submission and had just been delivered to our editors.

With the increase in demand, it became necessary to professionalize our group of revisers and during the national Meetings we started to hold workshops with the revisers to make the assessments more uniform. The results came quickly. The evaluation time fell substantially and although the number of published papers grew, causing an increase of 200 pages per year in the journal, the percentage of papers turned down went up to 35%.

Those were the conditions for our Journal to be indexed by ScIELO - revision papers - over 80 scientific papers published per year, 6 annual editions and national distribution. The indexation occurred readily, and ours was one of the first specialty journals to make it.

However, we still lacked an important step for visibility. Although SciELO supplied a search platform that could be reached by anyone in the world, the issues available were only the ones uploaded after the indexation date. It was necessary to recover the large collection of more than 60 years of our journal and we did this by scanning all the previous issues and inserting them in our recently-created web page.

Upon starting the scanning, we stumbled upon another bottleneck factor against the publishing flow. The growing number of submissions coming from all the states of the Brazilian Federation, and the large number of mail to and from two or three revisers created a huge pile of correspondence.

IT was the solution, together with using the internet to send and receive files, a system already in use by some of the main journals in the world. Because of the cost of foreign platforms at the time, we created a totally national publishing process. So efficient, that it started to be used by most of the other journals in the country in the years to come. With this, the process of paper submission, evaluation and approval dropped from 6 to 2 months.

With the guaranteed publishing flow, of course the quality of selected papers was increasing and the confidence that our journal's presence abroad could be tested was settled. The internet was a facilitator, but the culture of using paper was still prevalent in the academia. To receive greater attention from the academia, we needed to print a journal in paper, in English, which could be directly distributed to the libraries of the main universities and healthcare centers of the world. It was the beginning of the Brazilian Journal of Otorhinolaryngology (BJORL).

The way paved by the journal idealizers was kept with great difficulty by a persistent community, which matured substantially in the 80 years that passed. The journal only represented the steps of this community and we can say that today it walks relentlessly to achieve the same level of excellence of this community in the international setting.

**Figure 1:**
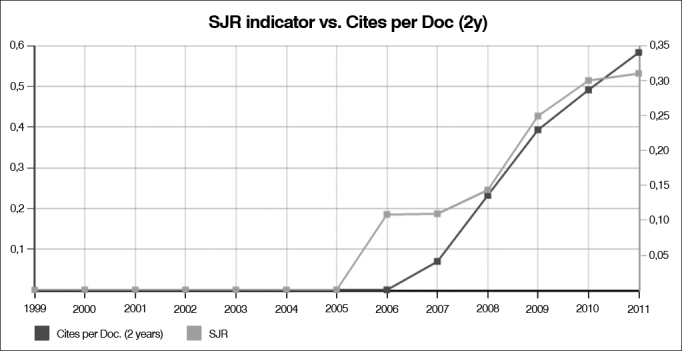
The curve shows the relevant growth in BJORL citations starting in 2005.

**Figure 2:**
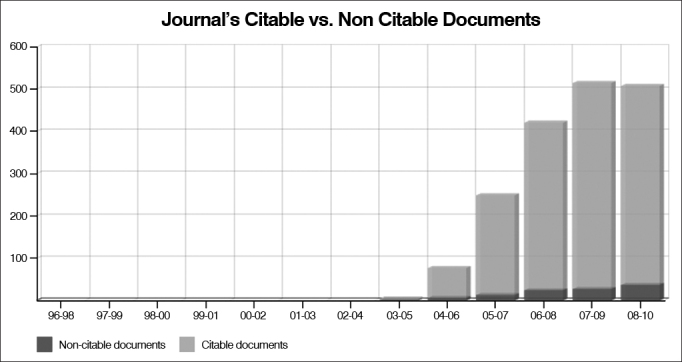
Ratio of citable papers (arising from research, papers presented in meetings and review papers) published in the BJORL.

In a recent comparative evaluation (2011) carried out by the SCImago Journal & Country Rank, a portal which includes the journals contained in the Scopus^®^ data base -one of most democratic within scientific journal bases in the world, the BJORL is considered the 49^th^ among the 93 international journals within the ENT umbrella (Table 1). It may seem feeble, but if you look at the journal's citation curve between 2005 to 2011 (http://www.scimagojr.com/journalsearch.php?q=4700152300&tip=sid&clean=0) (Figures 1 and 2), we can see that the BJORL had a soaring growth, projecting an impact factor (index that established the level of citation of the journals) similar to that of the 10 most traditional journals for the next 5 to 10 years.

**Table 1:** Scopus Journal Rank index of Otorhinolaryngology journals.

Rank	Journal	SJR	Total docs. (3 years)	Total citations (3 years)	Country
1	Audiology and Neurotology	1.688	151	397	Switzerland
2	Ear and Hearing	1.613	255	712	USA
3	Otology and Neurotology	1.396	766	1,478	USA
4	Advances in Oto-Rhino-Laryngology	1.046	44	84	Switzerland
5	International Journal of Audiology	1.042	342	592	England
6	Trends in Amplification	0.983	63	112	USA
7	Otolaryngology - Head and Neck Surgery	0.955	1,290	1,973	USA
8	Journal of Skull-Maxillo-Facial Surgery	0.947	265	454	USA
9	Head and Neck	0.936	677	1,566	USA
10	Journal of Voice	0.924	290	488	USA
11	Journal of the American Academy of Audiology	0.907	224	253	USA
12	Clinical Otolaryngology	0.903	557	405	England
13	Laryngoscope	0.862	1.891	2,889	USA
14	Dentomaxillofacial Radiology	0.786	246	386	England
15	Otolaryngologic Clinics of North America	0.785	285	517	England
16	Annals of Otology, Rhinology and Laryngology	0.781	449	616	USA
17	British Journal of Oral and Maxillofacial Surgery	0.779	663	934	USA
18	Current Opinion in Otolaryngology and Head and Neck Surgery	0.778	305	582	USA
19	Archives of Otolaryngology - Head and Neck Surgery	0.761	709	1,107	USA
20	International Journal of Oral and Maxillofacial Surgery	0.757	657	1,124	USA
21	International Journal of Pediatric Otorhinolaryngology	0.724	934	1,378	Italia
22	Acta Oto-Laryngologica	0.710	752	867	England

(Source: SCImago)

(Source: SCImago)

Not bad for an 80 year-old youngster. Congratulations!

